# Unveiling the Immunomodulatory Potential of Medicinal Plants: A Machine Learning Framework Coupled with Experimental Validation

**DOI:** 10.3390/life16101616

**Published:** 2026-09-26

**Authors:** Aicha Assouab, Hamza Tajmouati, Fatima Abdou-Allah, Khadija Akarid

**Affiliations:** 1 Biochemistry, Biotechnology and Immunophysiopathology Research Team, Health, Environment and Biotechnology Laboratory, Ain Chock Faculty of Sciences, Hassan II University of Casablanca (UH2C), Casablanca 20100, Morocco; aicha.assouab-etu@etu.univh2c.ma (A.A.); fatima.abdou-allah-etu@etu.univh2c.ma (F.A.-A.); 2 Iktos, Artificial Intelligence for New Drug Design, 75017 Paris, France; hamza.tajmouati@iktos.com

**Keywords:** machine learning, immunomodulation, medicinal plants, gene expression

## Abstract

Medicinal plants are known for their beneficial health effects through their ability to modulate the immune system, offering promising therapeutic options for various diseases. However, traditional methods for identifying these properties can be time-consuming, labor-intensive, and expensive. This study aims to (i) use machine learning (ML) to characterize the immunomodulatory effects of medicinal plants and (ii) validate the ML model via in vitro assays to assess the predicted immunomodulatory effects. To achieve this goal, data on medicinal plants were collected from the literature, structured and encoded using pre-trained language models to generate vector representations capturing semantic relationships among botanical, chemical, and microbiological entities. Several classifiers were benchmarked through a rigorous cross-validation strategy with optimal threshold tuning, and the best-performing model (logistic regression on BiomedBERT embeddings) reached a mean cross-validated F1-score of 0.787 ± 0.007 and, on the held-out test set (*n* = 37), an accuracy of 0.757, an F1-score of 0.743 and an AUC of 0.835; three of the four external validation plants were correctly classified. The results were validated experimentally using selected medicinal plants by assessing the modulation of inducible nitric oxide synthase (*iNOS)* and key inflammatory cytokine genes, including *IL-1β*, *IL-6*, and *TNF-α*. *C. spinosa* extract up-regulated *IL-1β* and *TNF-α* and down-regulated IL-6 transcripts in peritoneal macrophages, while *E. fragilis* extract induced a significant down-regulation of these cytokine genes and of the expression of *iNOS*. These preliminary results suggest that pre-trained biomedical embeddings provide a useful prioritization tool for investigating the immunomodulatory properties of medicinal plants.

## 1. Introduction

Nature is a rich and inspiring source of new remedies that significantly benefit humanity and enhance well-being. As a result, human health is closely intertwined with the use of traditional medicine. In recent years, the utilization of medicinal plants has expanded and increased worldwide as alternative therapies to prevent and treat various diseases and infections, driven by rising levels of toxicity and resistance to chemical treatments [1]. The medicinal properties of plants are attributed to the presence of a wide variety of secondary metabolites, including phenolics, terpenes, steroids, and alkaloids [2,3,4]. Due to their bioactive constituents, medicinal plants represent promising sources for developing effective antimicrobial agents targeting multidrug-resistant (MDR) pathogens while also exhibiting antioxidant, anti-inflammatory, and immunomodulatory properties [5,6].

Immunomodulation has garnered relevant academic interest due to implications for both basic research and clinical therapy [7]. The therapeutic potential of medicinal plants in modulating immune responses against infectious diseases and cancer is noteworthy. Immunomodulation involves modifying the immune response, including its induction, enhancement, or inhibition to maintain immune homeostasis and facilitate appropriate responses to various disorders [8]. Immunomodulators, which include both pharmaceuticals and bioactive compounds, can be classified into immunostimulatory and immunosuppressive types. A variety of bioactive compounds derived from plants have been shown to modulate immune responses by influencing cytokine production, activating immune cells, and regulating inflammatory pathways [9]. However, the investigation of medicinal plants as sources of bioactive compounds encounters several challenges, including lengthy methodologies and inefficiencies. Therefore, it is essential to advance ethnobotanical research as a preliminary step in this process by incorporating innovative approaches, such as artificial intelligence (AI). Traditional studies on the immunomodulatory effects of medicinal plants require significant time, effort, and financial resources. Moreover, the complex chemistry and their interactions with the immune system make it challenging to predict which compounds will have potent immunomodulatory activity [10].

Artificial intelligence (AI), particularly machine learning (ML), has revolutionized the identification of bioactive compounds by enabling high-accuracy predictions of biological activity with minimal human intervention [11,12,13]. Unlike traditional experimental methods, ML efficiently analyzes large datasets of plant-derived compounds, identifying potential immunomodulators through pattern recognition, feature selection, and predictive modeling. Techniques such as quantitative structure–activity relationship (QSAR) modeling, support vector machines (SVMs), random forests (RFs), and deep neural networks (DNNs) have been successfully employed to predict the immunomodulatory potential of phytochemicals based on their molecular structure and pharmacokinetic properties [14]. Furthermore, unsupervised learning and clustering algorithms facilitate the grouping of structurally similar bioactive compounds, aiding in the inference of their immune-related functions [15].

Various medicinal plants exhibit significant immunomodulatory effects, with ML playing a key role in elucidating their potential. For instance, ML-based virtual screening has validated the immune-regulating potential of *Panax ginseng* by predicting the interactions of its bioactive compounds with immune regulators and their modulation of critical inflammatory pathways [16]. Likewise, *Tinospora cordifolia* (guduchi) has been analyzed using ML models to predict its cytokine-modulating activity [17]. *Curcuma longa* (turmeric) has also been extensively studied using computational approaches combining molecular docking and ML to clarify its anti-inflammatory and immunomodulatory roles [18]. By integrating ML with cheminformatics, bioinformatics, and computational immunology, researchers can efficiently screen, classify, and validate compounds from medicinal plants, reducing costs and time while improving the accuracy of drug discovery.

A previous study explored classical ML pipelines with one-hot encoding for predicting the immunomodulatory character of medicinal plants, with an optimized SVM model achieving 73% accuracy [19]. However, one-hot encoding of categorical variables such as species names, metabolite content, and microorganism names fails to capture the rich semantic relationships between these entities. For instance, two closely related species (e.g., *Origanum compactum* and *Origanum elongatum*) receive orthogonal representations under one-hot encoding despite sharing taxonomic, chemical, and pharmacological similarities. To address this limitation, the present study proposes a novel approach based on pre-trained textual embeddings from language models. By serializing each sample into a structured text string and encoding it with a pre-trained transformer model, we obtain dense vector representations that capture domain-specific semantic relationships, including botanical taxonomy and chemical nomenclature.

This study emphasizes the significant contribution of ML to identifying the immunomodulatory properties of Moroccan medicinal plants, complemented by experimental validation. We specifically focused on the production and inhibition of key molecules, including nitric oxide (NO), generated through the activation of inducible nitric oxide synthase (*iNOS*). Furthermore, we assessed cytokine gene expression like *IL-1β*, *IL-6*, and *TNF-α*, which serve critical functions in the immune response to pathogenic infections.

## 2. Materials and Methods

### 2.1. Dataset

The dataset was systematically curated from an extensive review of over 100 peer-reviewed articles investigating the immunomodulatory properties of medicinal plants, as described in [19]. A total of 183 medicinal plants were selected and compiled into a structured dataset, incorporating key phytotherapeutic attributes: botanical species (SPECIES), geographical region (REGION), used plant part (USED PART), metabolite content (METABOLITE CONTENT), tested microorganism (TESTED MICROORGANISM), and effective concentration (EFFECTIVE CONCENTRATION). The target variable (TARGET) is binary, indicating whether the induction of *iNOS* and cytokine gene expression (class 1) or inhibition (class 0) was observed (Table 1).

The class distribution is relatively balanced with 82 positive samples (44.8%) and 101 negative samples (55.2%). Missing or undetermined values (noted as “ND”) were replaced with the string “Unknown” to preserve the information that the value is unavailable.

In addition to the 183 labeled samples used for model training and evaluation, a validation set of 4 plants with known immunomodulatory status (*Kalanchoe pinnata*, *Phyllanthus amarus*, *Eremanthus crotonoides*, and *Empetrum nigrum*) was used to verify the model’s predictions independently. Furthermore, 11 Moroccan medicinal plants with uncertain immunomodulatory effects were compiled as an external prediction set.

### 2.2. Data Preprocessing: Textual Serialization

Unlike the previous approach [19] which relied on one-hot encoding of categorical and continuous variables, we adopt a rich textual representation. Each sample is serialized into a structured text string that concatenates all six descriptors into a single sentence:

“Species: [SPECIES]. Region: [REGION]. Part: [USED PART]. Metabolites: [METABOLITE CONTENT]. Microorganism: [TESTED MICROORGANISM]. Concentration: [EFFECTIVE CONCENTRATION].”

This representation is intended to allow the embedding model to capture semantic relationships between variables, notably botanical taxonomy (e.g., plants from the same genus receive similar representations) and chemical nomenclature (e.g., “flavonoid” and “phenolic compound” are recognized as related).

### 2.3. Text Embeddings

The serialized text strings were transformed into dense numerical vectors using pre-trained sentence embedding models from the sentence-transformers library [20]. Two models were evaluated:

1. BiomedNLP-BiomedBERT (768 dimensions): a BERT-based model pre-trained on biomedical corpora (PubMed abstracts and PMC full-text articles) [21], capturing domain-specific pharmacological and biological semantics.
2. all-mpnet-base-v2 (768 dimensions): a general-purpose MPNet architecture with high semantic quality on the MTEB benchmark, serving as a non-domain-specific baseline [20].

Both textual embedders produce dense 768-dimensional vectors where semantically similar inputs are geometrically close. This is particularly advantageous for our small dataset (*n* = 183), as the model leverages knowledge from millions of biomedical texts during pre-training.

Implementation details. The domain-specific encoder was the public checkpoint microsoft/BiomedNLP-BiomedBERT-base-uncased-abstract-fulltext (Hugging Face Hub, revision e1354b7a3a09615f6aba48dfad4b7a613eef7062), a BERT-base architecture with 12 transformer layers and a hidden size of 768 [21]; the general-purpose baseline was sentence-transformers/all-mpnet-base-v2 [20]. Both checkpoints were loaded through the SentenceTransformer interface. Because the BiomedBERT checkpoint does not ship a sentence-transformers head, the library builds a Transformer module followed by a mean-pooling module over the token embeddings of the last hidden layer, restricted to non-padding tokens by the attention mask, with no normalisation layer; each serialized record is therefore mapped to a single 768-dimensional vector.

Tokenization used the uncased WordPiece tokenizer shipped with each checkpoint (vocabulary of 30,522 word pieces, do_lower_case = true) with a maximum sequence length of 512 tokens. The serialized records are between 26 and 173 word pieces long (median 58 over the 183 labelled records); so, no record was truncated. The encoder weights were kept frozen and used as a fixed feature extractor: embeddings were computed once under torch.no_grad(), without any fine-tuning, and only the standardisation, the PCA and the classifier were fitted, inside each cross-validation fold.

### 2.4. Dimensionality Reduction

To reduce overfitting risks due to the small sample size (*n* = 183) compared to the embedding dimensionality (768), Principal Component Analysis (PCA) was applied, retaining 95% of explained variance (yielding approximately 91 principal components). Embeddings were standardized before PCA using statistics (mean and standard deviation) estimated on the training set only. The full transformation chain (StandardScaler → PCA → Classifier) was encapsulated to ensure that each cross-validation fold fits transformations only on its training split, thereby preventing data leakage.

### 2.5. Classification Algorithms

Five supervised classifiers were compared, spanning both linear and non-linear model families (Table 2):

- Logistic Regression: linear model with L1 (Lasso) or L2 (Ridge) regularization.
- Linear SVM: linear-kernel support vector machine.
- RBF SVM: support vector machine with radial basis function kernel.
- Random Forest: ensemble of decision trees.
- XGBoost: gradient-boosted trees with tuneable depth and learning rate.

**Table 2:** Hyperparameter grids explored during cross-validated optimization.

Algorithm	C	Γ	n_Estimators	Max_Depth	Other Parameters
Logistic Regression	0.001–100 (11 values)	—	—	—	penalty ∈ {L1, L2}
SVM-Linear	0.001–100 (11 values)	—	—	—	—
SVM-RBF	0.1, 1, 10, 100	scale, auto, 0.001, 0.01, 0.1	—	—	—
Random Forest	—	—	50, 100, 200	3, 5, 7, None	min_samples_split ∈ {2, 5, 10} min_samples_leaf ∈ {1, 2, 4}
XGBoost	—	—	50, 100, 200	3, 5, 7	learning_rate ∈ {0.01, 0.1, 0.3} subsample ∈ {0.8, 1.0}

### 2.6. Validation Strategy and Model Selection

To ensure an unbiased and robust evaluation, we used a multi-split strategy with optimal threshold selection, improving upon the nested k-fold cross-validation used by Zerouali et al. [19].

**Robust cross-validation with multiple fold shuffles:** The data were split once into a fixed training set (80%, *n* = 146) and a held-out test set (20%, *n* = 37) with stratification. On the training set, 3-fold stratified cross-validation (CV) was repeated with five different fold shuffles to ensure that the model selection was robust to fold assignment. The results were aggregated across the five shuffles (mean ± standard deviation).

**Optimal threshold F1:** Instead of using the default classification threshold of 0.5, we adopted an optimal threshold approach: for each cross-validation fold, the classification threshold was determined by maximizing the F1-score on the validation fold over the range [0.05, 0.95]. This decouples the model’s probability calibration from the classification decision, allowing models with good discriminative capacity to be selected regardless of the default threshold. The mean optimal threshold across folds was used at test time.

**Final evaluation:** The best model (highest mean optimal-threshold CV F1 across the fivefold shuffles) was retrained on the full training set (*n* = 146) and evaluated once on the held-out test set (*n* = 37). This single evaluation avoids any test-set leakage into the selection process.

Software, random seeds and reproducibility. All analyses were run on CPU in float32 with Python 3.11.10, torch 2.10.0, transformers 4.57.6, sentence-transformers 5.2.2, scikit-learn 1.7.2, numpy 2.4.2, pandas 3.0.0 and xgboost 3.2.0; the exact versions are pinned in the lock file distributed with the code. Random seeds were fixed for every step that determines the selected model: 42 for the stratified train/test split, 42, 123, 456, 789 and 2024 for the five cross-validation fold shuffles, 42 for the randomized PCA solver, 42 for the logistic regression (solver lbfgs, deterministic) and 42 for the bootstrap generator. The random forest and two SVMs (with Platt scaling) were not seeded; so, their cross-validated F1-scores in Figure 1 may vary slightly (~0.01) between runs, without affecting the encoder ranking or the selected model. All scripts, data, embedding matrices, thresholds, and results are publicly available (see Data and Code Availability Statement) so that every number reported here can be regenerated end to end.

### 2.7. Evaluation Metrics

Performance was assessed with the following metrics: F1-score (at the optimal threshold), Average Precision (AP, area under the precision-recall curve), AUC (area under the ROC curve), Accuracy (at the optimal threshold), and Precision/Recall (at the optimal threshold). Optimal-threshold F1-score was used as the primary metric for model selection, while AP and AUC were reported as complementary ranking-oriented metrics.

#### Prediction Stability, Threshold Sensitivity and Probability Calibration

Due to predictions near the decision threshold, stability was assessed via a non-parametric bootstrap. The complete pipeline (standardisation, PCA at 95% explained variance, and logistic regression with fixed hyperparameters) was refitted on 1000 bootstrap resamples of the 183 labelled records. Each of the 15 unlabelled plants (4 validation and 11 Moroccan) was scored by every refitted model. The results include the point estimate, the 2.5th and 97.5th percentiles of the bootstrap distribution as a 95% confidence interval, and the proportion of resamples where the plant was called induction at t = 0.39.

Threshold sensitivity was assessed over the range of optimal thresholds actually selected by the five cross-validation fold shuffles (0.30, 0.38, 0.39, 0.43 and 0.44; mean 0.39). For every value in that range the predicted class of the 15 plants was recomputed and any change in call was recorded.

Probability calibration was evaluated using out-of-fold probabilities from ten repetitions of stratified 5-fold cross-validation on the 183 labelled records, reporting AUC, Brier score (and baseline), expected calibration error over five quantile bins, calibration slope obtained by logistic recalibration of the log-odds, and reliability diagram. Finally, the three nearest labelled records of each unlabelled plant were retrieved by cosine similarity in the embedding space in order to describe what the model matches.

### 2.8. Experimental Validation

#### 2.8.1. Plant Material and Extraction Procedure

Following ML prediction, *Capparis spinosa* (P1) and *Ephedra fragilis* (P2) were used for experimental validation. The aerial parts of P1 and P2 (leaves and stems) were collected according to the procedure described in [22,23]. Briefly, the aerial part of P1 was washed, air-dried, ground into a fine powder, and stored at 4 °C. Then, 10 g of powder was extracted with 100 mL of 70% ethanol (PROLAB, France) at room temperature for 24 h, filtered, and the solvent was evaporated using a rotary evaporator (Büchi Rotavapor R-200, New Castle, DE, USA) at 40 °C under reduced pressure. P2 was extracted with 62% ethanol (1/10, *w*/*v*) by maceration at 44.5 °C for 16 h, followed by the filtration and evaporation as described above. The resulting extracts were lyophilized and stored at −20 °C until use. The percentage of yielding extracts was calculated as follows:

Yield percentage = (weight of sample extract/initial weight of sample) × 100

#### 2.8.2. Determination of Total Phenolic and Flavonoid Content

Total phenolic content was determined using the Folin–Ciocalteu method [24]. Specifically, 0.5 mL of extract was reacted with 2 mL of Na_2_CO_3_ (75 g·L^−1^) (Merck Life Science, Darmstadt, Germany) and 2.5 mL of 10% (*v*/*v*) Folin–Ciocalteu reagent (Sigma-Aldrich, St Louis, MO, USA). After 30 min at room temperature, the absorbance was read at 725 nm using a UV–vis spectrophotometer (model Biomate 3, Madison, USA).

Total flavonoid content was determined using the Aluminium chloride colorimetric method [25]. Here, 0.5 mL of extract or standard was mixed with 0.5 mL of 2% AlCl3-ethanol solution, incubated for 1 h at room temperature, then the absorbance was measured at 420 nm.

Results were expressed as mg of Gallic acid equivalents (GAE) or quercetin equivalents (QE) per gram of dry plant material weight (dw), respectively. All analyses were performed in triplicate.

#### 2.8.3. Cell Culture and Cytotoxicity Assay

The effects of P1 and P2 extracts on peritoneal macrophages viability were assessed using the MTT assay [26]. Macrophages were treated with different concentrations of P1 (25–400 μg/mL) and P2 (12.5–200 μg/mL) extracts for 72 h. After incubation, 10 µL of MTT solution (5 mg/mL; Sigma, St Louis, MO, USA) was added to each well and incubated for an additional 4 h. The resulting formazan crystals were dissolved with 100 μL of DMSO per well, and absorbance was measured at 570 nm using a microplate reader. Cultured macrophages without extracts were used as a control. The experiment was performed in triplicate across three independent runs. IC_50_ values were calculated using the Fit Spline/LOWESS method in GraphPad Prism software version 8.0.

#### 2.8.4. *INOS* and Cytokine Gene Expression

Peritoneal macrophages were cultured overnight at 37 °C at a density of 5 × 10^5^ cells per well. After a 2 h pretreatment with non-cytotoxic concentrations of P1 (100 μg/mL) and P2 (25 μg/mL) hydroethanolic extracts, the cells were stimulated with or without IFN-γ (10 ng/mL) and Lipopolysaccharide (LPS) (50 ng/mL). Supernatants collected 24 and 48 h post-stimulation for nitrite assays, and cell pellets were reserved for molecular assay.

Total RNA was extracted from macrophages, using TRIZURE reagent (Bioline, Luckenwalde, Germany) and conserved at −80 °C until performing RT-qPCR analysis with 4X CAPITAL™ 1-Step qRT-PCR Probe Master Mix (Biotechrabbit, Berlin, Germany). The PCR was conducted in 20 μL reaction mixtures; 5 μL of 4X CAPITAL qPCR Probe master Mix, 0.8 µL of each primer (10 µM), 1 µL of reverse transcriptase, 3 µL of RNA (6 ng/µL), and 9.4 µL Rnase-Free water. Primer Sequences are shown in Table 3. RT-qPCR conditions were 50 °C for 10 min (reverse transcription), 95 °C for 3 min (polymerase activation), and 40 cycles of 95 °C for 10 s (denaturation), 65 °C for 30 s (annealing/extension). Real-time fluorescence detection was performed using BioRad CFX96 Real-time PCR Thermal Cycle (BioRad, Marnes-la-Coquette, France). The results were calculated using the 2^^−ΔΔCt^ relative quantification method normalized to housekeeping gene (β-actin).

### 2.9. Statistical Analysis

Data are represented as mean ± SEM. The significance of the results was calculated using *t*-test and one-way ANOVA analysis of variance with Tukey’s post hoc test for multiple comparisons was implemented using GraphPad Prism version 8.0. The threshold of significance was fixed at *p* < 0.05.

## 3. Results

### 3.1. Model Performance

Figure 1 reports the model selection results aggregated over fivefold shuffles for all 10 tested combinations (2 embeddings × 5 classifiers), sorted by decreasing mean CV F1 (optimal threshold). BiomedBERT embeddings consistently outperformed all-mpnet-base-v2 across all classifiers (mean CV F1 gap: +0.05), confirming the advantage of domain-specific pre-training for pharmacological prediction tasks.

The Logistic Regression + BiomedNLP-BiomedBERT model was selected as the best model, with a mean cross-validated F1-score of 0.787 ± 0.007, exhibiting the highest mean performance and the lowest variance across fold shuffles. This model combines a simple linear classifier (C = 0.001, L2 regularization) with domain-specific biomedical embeddings pre-trained on PubMed and PMC corpora. The optimal classification threshold, determined during cross-validation, was 0.39.

The model was retrained on the full training set (*n* = 146) and evaluated once on the held-out test set (*n* = 37). Table 4 reports the test set performance and comparison with the previous SVM baseline [19].

The textual embedding approach achieved a test accuracy of 0.757 and an F1-score of 0.743, representing respective gains of +2.7% and +9.3% over the one-hot encoding + SVM baseline [19] while relying on a simpler linear classifier (Logistic Regression). However, given the limited test set size (*n* = 37), these improvements should be interpreted with caution.

### 3.2. Application to Moroccan Medicinal Plants

The selected model (Logistic Regression + BiomedBERT, C = 0.001) was retrained on 100% of the labeled data (*n* = 183) and applied to the validation set (4 plants) and Moroccan prediction set (11 plants) using the optimal threshold (t = 0.39) (Table 5).

The model predicted, at the operating threshold t = 0.39, 7 out of 11 Moroccan plants as predicted induction representing 63.6% of the total. Among the highest-ranked plants, *Capparis spinosa* (P1, P = 0.414) was predicted as induction, whereas *Ephedra fragilis* (P2, P = 0.352) received a borderline predicted inhibition, close to the optimal threshold (Table 6).

On the validation set, three of the four plants were correctly classified. The misclassified plant, *E. crotonoides*, inhibits NO and TNF-α production but scored of 0.437, that is 0.047 above the threshold and well inside the unstable band identified by the bootstrap (77% of resamples). *Empetrum nigrum* was correctly classified in only 52% of the resamples. An agreement of 3 out of 4 on a set of this size should therefore not be read as an estimate of external accuracy.

Since several scores are close to the decision threshold, bootstrap resampling was used to assess call stability (Section Prediction Stability, Threshold Sensitivity and Probability Calibration). The resulting intervals and call frequencies are reported in Table 5 and Table 6, with the full bootstrap distributions in Table S1 and Figure S1A. Only three of the eleven Moroccan plants showed stable calls in over 95% of resamples: *M. communis* (induction in 100% of resamples), *S. clandestina* (99.5%) and *C. sativa* (inhibition in 99% of resamples). *O. elongatum* was stable at 84%. The remaining seven plants have bootstrap intervals that straddle the threshold, including the two species taken forward to the bench: *C. spinosa* is called induction in 69% of the resamples and *E. fragilis* in 38%; so, both are borderline rather than confident predictions.

Sensitivity to the decision threshold (0.30 to 0.44) was assessed using the optimal thresholds from five-fold shuffles. The number of Moroccan plants called induction ranges from 10 of 11 at t = 0.30 to 3 of 11 at t = 0.44. Only six of the fifteen unlabelled plants (*M. communis*, *S. clandestina*, *O. elongatum*, *C. sativa*, *K. pinnata* and *P. amarus*) maintained the same call over the whole band. *C. spinosa* switches to inhibition at t ≥ 0.42, and *E. fragilis* switches to induction at t ≤ 0.35. The counts at each threshold are given in Table S2.

Probability calibration was evaluated using out-of-fold predictions from ten repetitions of stratified 5-fold cross-validation (*n* = 183). The model discriminates moderately (AUC 0.820 ± 0.011) with probabilities better than just using the class prior (Brier score 0.179 ± 0.004 vs. 0.247). However, the model is under-confident: the calibration slope is 1.60, and the expected calibration error is 0.086, mainly due to the strong L2 penalty (C = 0.001) that shrinks scores toward the prior of 0.45. That compression also explains why the F1-optimal threshold is 0.39 rather than 0.5. The reliability diagram (Figure S1B) is not monotonic in the middle of the scale: the 0.26 to 0.37 bin has an observed induction rate of 0.08, below the adjacent bins. The values reported in Table 5 and Table 6 should therefore be read as model scores used to rank candidates for experimental follow-up, and not as calibrated biological probabilities.

To characterise what drives the predictions, the three nearest labelled records of each unlabelled plant were inspected in the raw embedding space (Table S3). These neighbours do not explain the calls. For *M. communis* they carry mixed labels (*Rhus tripartitum*, inhibition; *Morinda citrifolia* and *Larrea divaricata*, induction), whereas for *W. saharae* and *E. altissima* all three neighbours are inhibition records although both plants are predicted induction. For the plants tested against bacteria, and for those with no reported microorganism, the neighbours are mainly records that share this field (bacterial targets or “ND”) rather than taxonomically related plants, and all similarities lie between 0.992 and 0.999. The raw embedding space is thus strongly anisotropic, and the discrimination comes from the PCA-whitened linear model rather than from an obvious taxonomic or chemical look-alike.

A limitation concerns the records with incomplete descriptors. *C. spinosa*, *E. fragilis* and *E. echinus* in the Moroccan set, together with *Empetrum nigrum* in the validation set, all carry the value “Unknown” for the tested microorganism and/or concentration, and cluster together in the principal component map (Figure 2A). Their score partly reflect shared missing data, rather than plant-specific information, highlighting a limitation of the text-serialization approach.

### 3.3. Biological Results Using Plants Predicted by the Model

According to the predictions generated by the machine learning model, *Capparis spinosa* (P1) was among the candidates predicted as induction (P = 0.414, 95% bootstrap interval 0.273–0.597) and *Ephedra fragilis* (P2) among those predicted as inhibition (P = 0.352, 0.238–0.540), both close to the decision threshold. Their exact ranks are not stable: across bootstrap resamples the rank of *C. spinosa* among the eleven Moroccan plants ranges from 2 to 10 (mean 5.6) and that of *E. fragilis* from 4 to 11 (Figure 2C). Both were therefore taken forward as borderline candidates rather than as confident predictions, and on grounds of availability. *Capparis spinosa* was chosen due to its widespread occurrence in Morocco and its considerable economic value, while *Ephedra fragilis* was selected for its relevance and availability in the laboratory setting.

#### 3.3.1. Phytochemical Analysis of Studied Plants

The extraction yields of the aerial parts of *C. spinosa* and *E. fragilis* were evaluated to assess the impact of extraction conditions using a hydroethanolic solvent. The *C. spinosa* hydroethanolic extract (CSE) yielded the highest percentage at 12.9%, while the *E. fragilis* extract (EFE) yielded 8.33%. The total phenolic and flavonoid levels in CSE and EFE were measured, revealing that the hydroethanolic extracts of the studied plants contained polyphenols and flavonoids at varying concentrations (Table 7).

#### 3.3.2. Effects of *E. fragilis* and *C. spinosa* Extracts on Cell Viability

The cytotoxicity of hydroethanolic extracts of CSE and EFE was assessed using the MTT assay on peritoneal macrophages. The results showed that CSE did not affect cell viability at concentrations ranging from 25 to 400 μg/mL. In contrast, EFE exhibited non-cytotoxic effects solely at concentrations of 12.5 μg/mL and 25 μg/mL (Figure 3).

#### 3.3.3. Cytokine and *INOS* Gene Expression

Our results showed that peritoneal macrophages treated with CSE exhibit an upregulation of *IL-1β* gene expression 24 h post-treatment, even in the absence of LPS stimulation (Figure 4A). Furthermore, the levels of *TNF-α* significantly increased in response to stimulation, while *IL-6* exhibited a marked decrease in the presence of LPS (Figure 4B,C).

EFE exhibited a downregulation of *IL-1β*, *IL-6*, and *TNF-α* expression in unstimulated macrophages. However, under conditions of stimulation, the difference induced by EFE was not statistically significant (Figure 5A–C). 

CSE and EFE did not affect NO levels at 24 and 48 h post-treatment, regardless of LPS and IFN-γ stimulation. This lack of nitric oxide synthesis is not attributable to the downregulation of *iNOS*, given that no statistically significant variations in *iNOS* expression were observed, except for EFE, which showed downregulation at 24 h post-treatment without stimulation (Figure 4D and Figure 5D).

## 4. Discussion

In the current study, we developed an improved machine learning predictive model to assess the immunomodulatory potential of medicinal plants, advancing beyond the traditional one-hot encoding approach [19]. The key methodological innovation is the use of pre-trained biomedical textual embeddings (BiomedNLP-BiomedBERT) to represent plant samples, replacing the traditional one-hot encoding of categorical variables. This approach leverages the semantic knowledge embedded in large-scale biomedical language models and, unlike one-hot encoding, provides a representation for species and descriptors absent from the training data.

The Logistic Regression + BiomedBERT model achieved a test F1-score of 0.743, which is higher than the F1-score of 0.65 quoted for the SVM baseline (F1 = 0.65) [19]. Notably, this result was obtained with a simpler classifier (Logistic Regression vs. SVM with RBF kernel), suggesting that the discriminative power resides primarily in the quality of the textual embeddings rather than in the complexity of the classifier. The domain-specific BiomedBERT embeddings consistently outperformed the general-purpose mpnet embeddings across all five classifiers (mean CV F1 gap: +0.05), suggesting that pre-training on biomedical corpora captures relevant pharmacological semantics for this task. Two caveats apply to the size of this gain. It rests on a single held-out split of 37 records and on a figure reported in a different study; so, it is indicative rather than established; and under repeated 10 × 5-fold cross-validation the expert-coded one-hot features perform on a par with the embeddings on the labelled set. The advantage we can defend is therefore not a large accuracy gain but the applicability of the embeddings to species, plant parts and descriptors absent from the training vocabulary, for which the one-hot representation collapses to the class prior.

When applied to 11 Moroccan medicinal plants, 7 species were predicted as predicted induction at the optimal threshold (t = 0.39), with *Myrtus communis* (P = 0.588) and *Salvia clandestina* (P = 0.538) receiving the highest probabilities. The experimental validation of *Capparis spinosa* (predicted induction P = 0.414) and *Ephedra fragilis* (borderline inhibition P = 0.352) produced changes in cytokine gene expression (*IL-1β*, *TNF-α*, *IL-6*) in peritoneal macrophages that are consistent with an immunomodulatory action. For *C. spinosa* the response was mixed, with up-regulation of *IL-1β* and *TNF-α* and down-regulation of *IL-6*, while *E. fragilis* significantly downregulated these genes. These preliminary observations on two species, both of which received borderline scores, cannot establish the predictive accuracy of the model.

Our study showed that the hydroethanolic extract of *C. spinosa* (capers) modulates macrophage activation by upregulating *IL-1β* and *TNF-α* gene expression. This plant, part of the Capparaceae family, is rich in secondary metabolites including polyphenols and flavonoids [42]. The phytochemical profile of plants, including *C. spinosa*, is influenced by various factors, such as geographical region, the part of the plant used and extraction methods [23,43,44]. Previous research has shown that *C. spinosa* extracts stimulate NO production by macrophages in vitro, thereby inhibiting the proliferation of *Leishmania* parasites [23]. Additionally, a study demonstrated that the methanol extract of *C. spinosa* buds exhibits antiviral and immunomodulatory effects on peripheral blood mononuclear cells (PBMC) by upregulating pro-inflammatory Th1 cytokines such as IL-12, IFN-γ and TNF-α [45]. Moreover, *C. spinosa* extracts exhibit immunomodulatory and anti-inflammatory properties by either upregulating or downregulating the expression of pro-inflammatory cytokine genes, including *IL-17*, *IL-1β*, *TNF-α*, *IL-6*, which are involved in inflammatory disorders such as rheumatoid arthritis and atherosclerosis [46,47,48,49]. The discrepancies between these findings and our results may be attributed to several factors, including the use of different plant parts, the specific cell types employed in the analyses, and the geographical origin of the plants. Regarding the highest-ranked plant in our predictive model, a Moroccan study showed that a fraction derived from *Myrtus communis* exhibits significant antioxidant and anti-inflammatory properties [50]. Moreover, *M. communis* from Iran has been documented to reduce inflammatory gene expression, like *COX-2* and *iNOS*, in LPS-stimulated J774.A1 macrophages [51]. Although the anti-inflammatory effects of *M. communis* are well documented, the immunomodulatory effects of the Moroccan species remain to be fully investigated.

Among the classified plants, *E. fragilis,* a member of the Ephedraceae family, is mainly found in North Africa, Europe, Asia and America [52]. Our results demonstrate that the hydroethanolic extract of *E. fragilis* exhibits anti-inflammatory effects on peritoneal macrophages by downregulating *IL-1β*, *TNF-α*, and *IL-6* gene expression. Both in vitro and in vivo studies showed that *E. fragilis* exhibits antioxidant, antiglycation and anti-inflammatory activities, attributed to their bioactive compounds such as polyphenol, flavonoids, and alkaloid, particularly ephedrine, which has immunostimulant properties [22,53,54].

Several limitations should be considered when interpreting these findings. Our machine learning model was trained on a limited, heterogeneous dataset extracted from the literature, which varies in experimental conditions, features, and parameters used to predict immunomodulatory activity. Additionally, the binary classification of induction or inhibition may not capture the complex immune responses a plant can elicit through other immune markers. Further experimental validation, involving different extracts, cell types, and plant species, is necessary to confirm these predictions and enhance the robustness and applicability of the proposed approach.

Given this, our predictive model is built using medicinal plant species that modulate NO and cytokine gene expression in response to diverse pathogens, which constitutes a significant proportion of our dataset. Most prior research aligns with our findings in the context of infection, which is consistent with the classifications established by our predictive model. Nevertheless, machine learning algorithms are data-intensive and typically require large amounts of training data [11]. Considering our currently available data, we explored various algorithms to identify those offering best results through enhanced performance. Similarly, a study showed the possibility to predict the anti-inflammatory effect of polymers, based on a restricted dataset [55]. Thus, our work suggests that even small but well-designed datasets can provide innovative approaches, promoting the utilization of phytochemical constituents derived from medicinal plants as immunomodulators.

## 5. Conclusions

In conclusion, we found that pre-trained biomedical textual embeddings improve the prediction of the immunomodulatory activity of medicinal plants compared to the traditional one-hot encoding approach. The Logistic Regression + BiomedBERT model achieved a test F1-score of 0.743 and AUC of 0.835, outperforming the previous SVM baseline by +9.3% in F1-score and +2.7% in accuracy on this single-test split; given the size of the split, and the fact that expert-coded one-hot features remain competitive under repeated cross-validation, this difference should be treated as preliminary. The semantic richness of biomedical embeddings, which are pre-trained on millions of PubMed articles, appears mainly useful in allowing unseen species and descriptors to be scored at all, which is not possible with one-hot encoding, and is therefore of interest for small datasets where traditional feature engineering is limited.

Our study is based on a limited dataset of 183 samples, which is suitable for certain analyses. However, expanding the dataset could improve the model’s generalizability to other contexts or species of medicinal plants, thereby enhancing its applicability and predictive power. The moderate overall performance highlights the difficulty of predicting immunomodulatory activity from botanical and chemical descriptors alone, without direct knowledge of the observed biological effect. Future work could explore data augmentation strategies, integration of molecular fingerprints from chemical databases, fine-tuning of the language model on domain-specific immunology corpora, or larger datasets to further improve predictive power. The probability-ranked list of Moroccan plants provides a prioritized guide for future experimental validation of immunomodulatory activity. Efforts to gather more diverse data will enhance the validity of predictive models and improve our understanding of the immunomodulatory properties of medicinal plants.

## Figures and Tables

**Figure 1:**
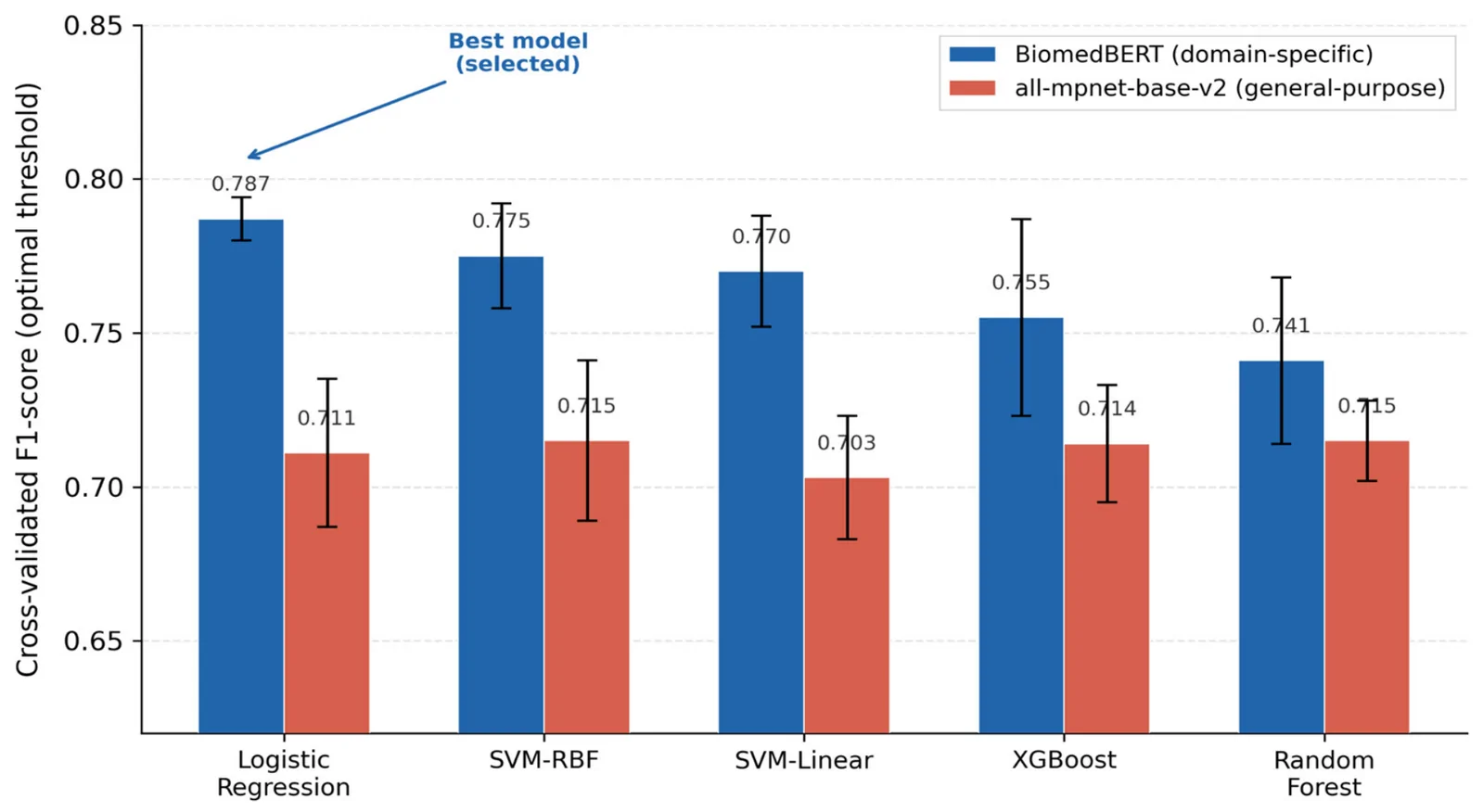
Cross-validated F1-scores (optimal threshold) for five classifiers using BiomedBERT (domain-specific) and all-mpnet-base-v2 (general-purpose) embeddings. Error bars represent ±1 standard deviation across fivefold shuffles.

**Figure 2:**
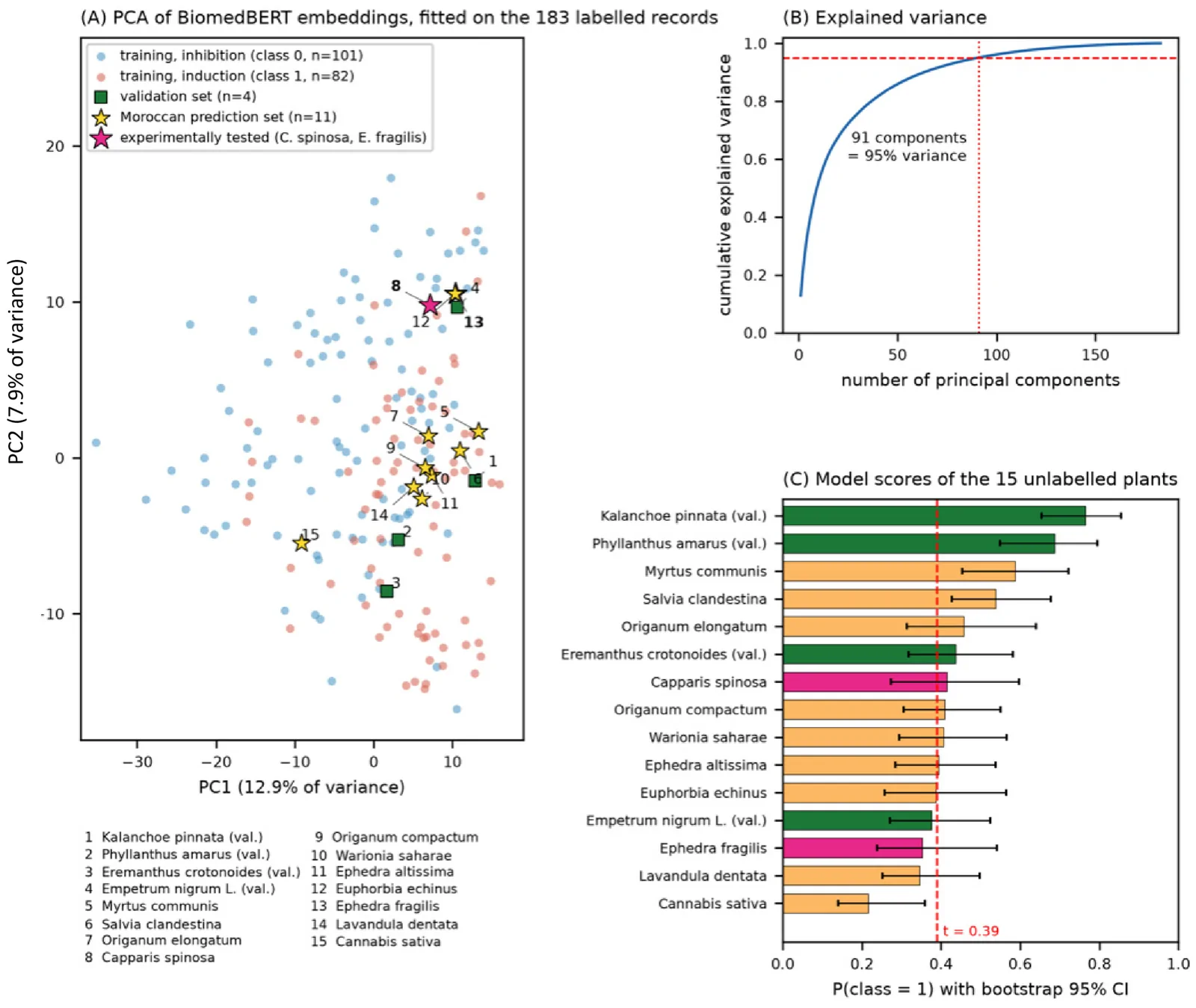
Principal component map of the BiomedBERT embedding space. (**A**) First two principal components of the standardized embeddings fitted on the 183 labelled records, coloured by class, with the 4 validation plants and the 11 Moroccan plants projected into the same space and *C. spinosa* and *E. fragilis* highlighted. (**B**) Cumulative explained variance; 91 components are required to reach 95%. (**C**) Model scores of the 15 unlabelled plants with their 95% bootstrap intervals and the decision threshold t = 0.39. PC1 and PC2 together carry only 20.8% of the variance; so, panel (**A**) is descriptive and the classifier operates on the 91 retained components; the two classes overlap substantially in two dimensions, which is consistent with the moderate AUC reported in Section 3.1. Unlabelled plants are numbered as in the key below panel (**A**). *E. fragilis* (13) and *E. echinus* (12) are projected onto almost the same point, as both records lack the tested microorganism and the effective concentration.

**Figure 3:**
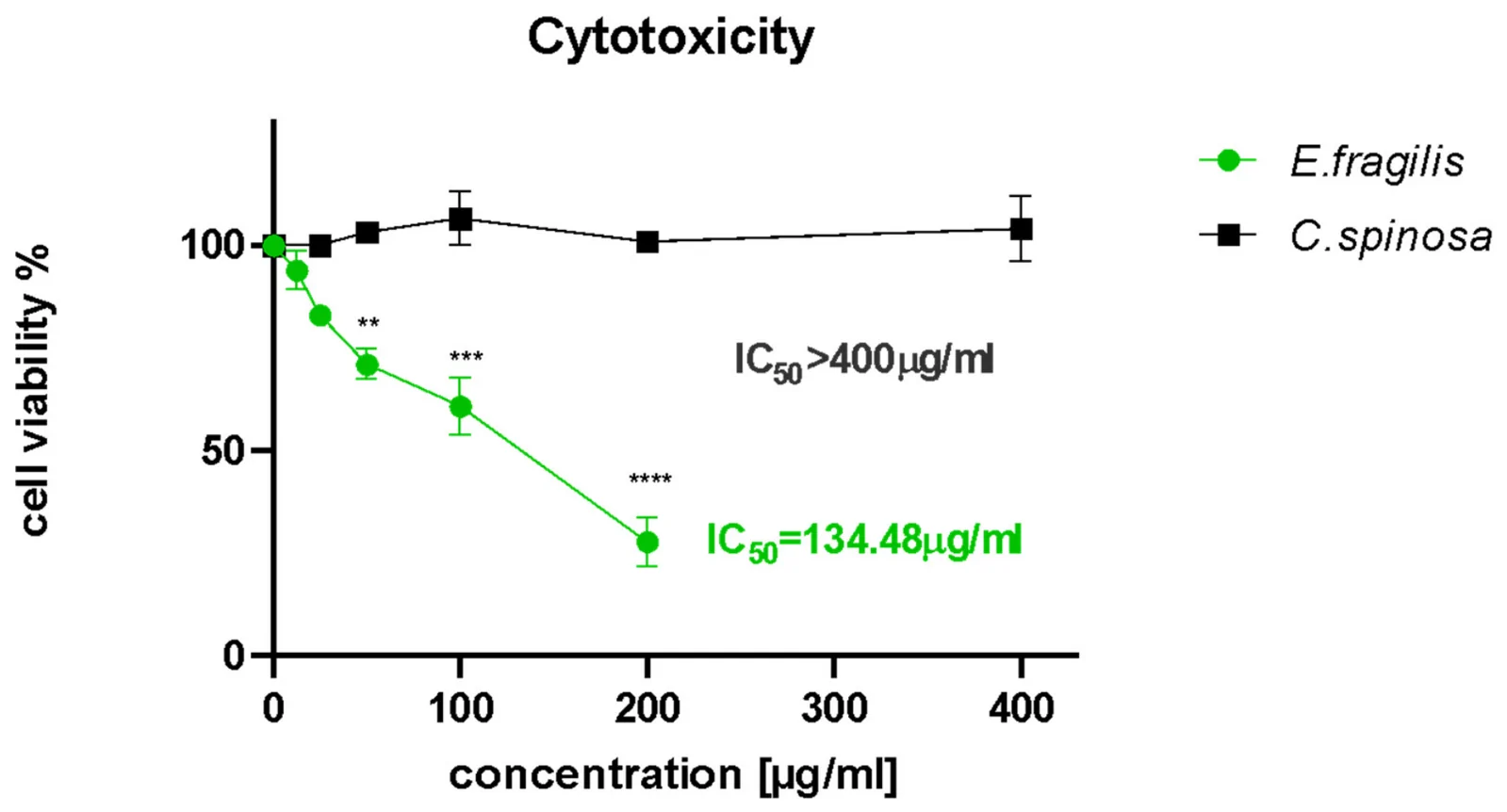
Effect of *C. spinosa* and *E. fragilis* extracts on the peritoneal macrophages’ viability. The cells were treated with CSE and EFE for 72 h. The values represent the mean ± SEM of three independent experiments. The IC_50_ is the concentration required to reduce cell viability by 50%. ** *p* ≤ 0.05, *** *p* ≤ 0.01, **** *p* ≤ 0.001.

**Figure 4:**
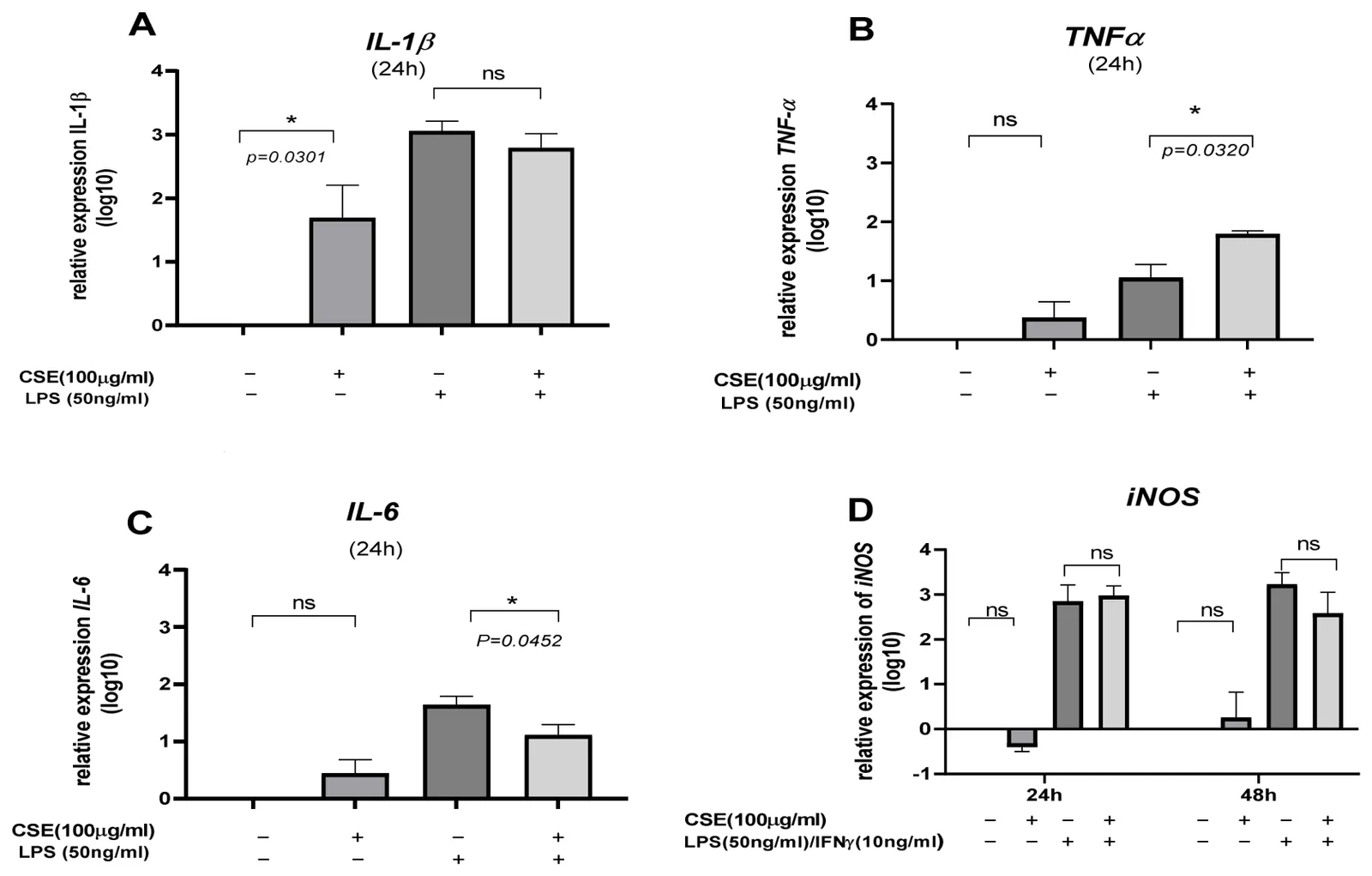
Effects of CSE at concentrations of 100 µg/mL on the expression levels of *IL-1β*, *TNF-α*, *IL-6* (**A**–**C**) and *iNOS* (**D**) in both stimulated and non-stimulated peritoneal macrophages. Differences between groups were assessed using unpaired *t*-test for the panel (**A**–**C**) and one-way ANOVA followed by Tukey’s multiple comparisons test for the panel (**D**). The values represent the mean ± SEM of three independent experiments. ns: not significant, * *p* ≤ 0.05.

**Figure 5:**
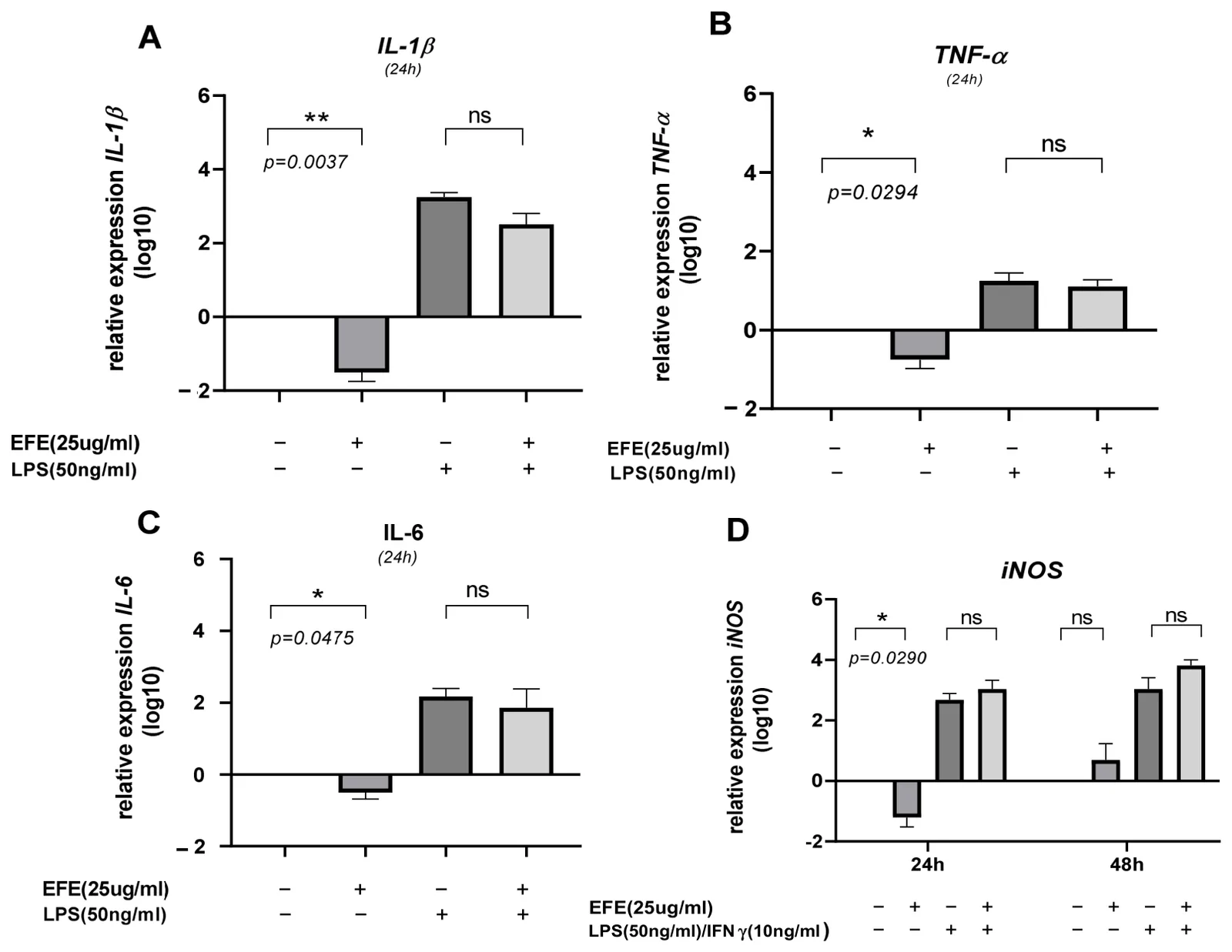
Effects of EFE at concentrations of 25 µg/mL on the expression levels of *IL-1β*, *TNF-α*, *IL-6* and *iNOS* in both stimulated and non-stimulated peritoneal macrophages. The values represent the mean ± SEM. Differences between groups were assessed using unpaired *t*-test for the panel (**A**–**C**) and one-way ANOVA followed by Tukey’s multiple comparisons test for the panel (**D**). The values represent the mean ± SEM of three independent experiments. ns: not significant, * *p* ≤ 0.05, ** *p* ≤ 0.01.

**Table 1:** Structure of the raw dataset.

Feature	Type	Description
SPECIES	Categorical	Botanical species name
REGION	Categorical	Geographical region of collection
USED PART	Categorical	Plant part used (leaf, root, stem, etc.)
METABOLITE CONTENT	Categorical	Phytochemical profile (flavonoids, alkaloids, etc.)
TESTED MICROORGANISM	Categorical	Target microorganism tested
EFFECTIVE CONCENTRATION	Continuous	Effective dosage concentration
TARGET	Binary (0/1)	Immunomodulatory activity (dependent variable)

**Table 3:** Primer for qRT PCR.

Primers	Forward	Reverse
*β-actin*	5′-CATTGCTGACAGGATGCAGAAGG-3′	5′-TGCTGGAAGGTGGACAGTGAGG-3′
*IL-1β*	5′-AGTTGACGGACCCCAAAAG-3′	5′-AGCTGGATGCTCTCATCAGG-3′
*INOS*	5′-CAGCTGGGCTGTACAAACCTT-3′	5′-CATTGGAAGTGAAGCGTTTCG-3′
*IL-6*	5′-TCCTACCCCAATTTCCAATGC-3′	5′-TGAATTGGATGGTCTTGGTCC-3′
*TNF-α*	5′-ATGAGAAGTTCCCAAATGGCC-3′	5′-CCACTTGGTGGTTTGCTACGA-3′

**Table 4:** Performance of the best model on the held-out test set (*n* = 37).

Metric	Value	vs. Baseline [19]
Accuracy	0.757	+2.7%
F1-score	0.743	+9.3%
Precision	0.722	—
Recall	0.765	—
Average Precision (AP)	0.865	—
AUC	0.835	—

**Table 5:** Predictions on the validation set (threshold t = 0.39), with the reported effect, the reference label, the experimental conditions and the citation of each source study, the agreement between the reference label and the prediction, and the stability of the call over 1000 bootstrap resamples of the training set.

Species	Region	Used Part	Conditions (Microorganism, Concentration) and Citation	Reported Effect (Label)	Call (t = 0.39)	P	95% CI	Induction Calls (%)	Agreement
*Kalanchoe pinnata*	Brazil	Leaf	*Leishmania chagasi* (parasite); in vivo, 400 mg/kg; [27]	Production of NO (1)	Induction	0.765	0.654–0.854	100	Yes
*Phyllanthus amarus*	India	Aerial parts	*Leishmania donovani* (parasite); in vivo, 5 and 10 mg/kg/day; [28]	Production of NO, IFN-γ and ROS (1)	Induction	0.687	0.549–0.795	100	Yes
*Eremanthus crotonoides*	Brazil	Leaf (crude extract)	*Mycobacterium tuberculosis* H37Rv (ATCC 27294); IC50 < 0.8 µg/mL (NO) and 5.83 ± 1.40 µg/mL (TNF-α); [29]	Inhibition of NO and TNF-α production (0)	Induction	0.437	0.318–0.582	77	No
*Empetrum nigrum* L.	Korea	Aerial parts	Microorganism not reported; 100 and 200 µg/mL; [30]	Inhibition of NO and TNF-α production (0)	Inhibition	0.376	0.271–0.524	48	Yes

P is the model score P (class = 1); 95% CI is the 95% bootstrap interval of that score. Reference labels follow the convention of Section 2.1 (1 = induction, 0 = inhibition of *iNOS* and cytokine gene expression). “Induction calls” is the percentage of the 1000 bootstrap resamples in which the plant was assigned to the induction class at t = 0.39. Agreement compares the reference label with the prediction; overall agreement is 3 of 4.

**Table 6:** Predictions on Moroccan medicinal plants (threshold t = 0.39), sorted by P (class = 1), with the experimental conditions and the citation of each source study and the stability of the call over 1000 bootstrap resamples of the training set.

Species	Region	Used Part	Microorganism	Concentration	Citation	Call (t = 0.39)	P	95% CI	Induction Calls (%)
*Myrtus communis*	Morocco	Leaf	Parasite	IC50 303 to 500 µg/mL	[31]	Induction	0.588	0.454–0.721	100
*Salvia clandestina*	Morocco	Whole plant	Parasite	IC50 > 1000 µg/mL	[32]	Induction	0.538	0.427–0.678	99.5
*Origanum elongatum*	Morocco	Leaf, flower	Virus (murine norovirus MNV-1)	Effective concentration 2%	[33]	Induction	0.458	0.314–0.640	84
*Capparis spinosa*	Morocco	Leaf/aerial part	Not reported	Not reported	[34,35]	Induction	0.414	0.273–0.597	69
*Origanum compactum*	Morocco	Aerial parts	Parasite	IC50 0.02 to 0.72 µg/mL	[36]	Induction	0.409	0.305–0.550	68
*Warionia saharae*	Morocco	Aerial parts	Bacteria	MIC 0.039 to 6.25 mg/mL	[37]	Induction	0.406	0.295–0.566	65
*Ephedra altissima*	Morocco	Root	Bacteria	MIC 4.00 and 16.00 mg/mL	[38]	Induction	0.394	0.285–0.537	58
*Euphorbia echinus*	Morocco	Whole plant	Not reported	Not reported	[39]	Inhibition	0.387	0.257–0.564	55
*Ephedra fragilis*	Morocco	Aerial parts	Not reported	Not reported	[22]	Inhibition	0.352	0.238–0.540	38
*Lavandula dentata*	Morocco	Aerial parts	Bacteria	MIC 0.041 to 0.338 mg/mL	[40]	Inhibition	0.345	0.252–0.497	34
*Cannabis sativa*	Morocco	Aerial parts	Pseudomonas aeruginosa DSM 50090 (bacteria)	MIC 1.2 to 37.8 mg/mL	[41]	Inhibition	0.215	0.139–0.359	1

P is the model score P (class = 1); 95% CI is the 95% bootstrap interval of that score. “Induction calls” is the percentage of the 1000 bootstrap resamples in which the plant was assigned to the induction class at t = 0.39. Tested microorganism and effective concentration are shown as encoded in the model input (dataset workbook), with harmonised spelling and units; “Not reported” corresponds to the values “Unknown” or “ND”.

**Table 7:** Total polyphenols and flavonoid content in *C. spinosa* and *E. fragilis* crude extracts.

	Total Polyphenols Content (mg GAE/g of _dw_) ^1^	Total Flavonoid Content (mg QE/g of _dw_) ^2^
*Capparis spinosa*	0.136 ± 0.008	0.055 ± 0.002
*Ephedra fragilis*	0.078 ± 0.008	0.029 ± 0.001

^1^ Presented as milligrams of gallic acid equivalents (GAE) per gram of dry plant material. ^2^ *Presented as milligrams of* quercetin (QE) per gram of dry plant material. Values are expressed as mean ± SD of triplicate measurements.

## Data Availability

The dataset generated and analyzed for this study is publicly available on Zenodo at https://doi.org/10.5281/zenodo.19451645. This concept DOI resolves to the latest version of the record (CC-BY 4.0), which also contains: the analysis scripts for embedding generation, model selection and thresholding, final prediction, bootstrap stability and calibration, and the principal component map, together with a pinned dependency list; the serialized text records and the BiomedBERT and mpnet embedding matrices; the record-level references for the 183 training rows, the 4 validation plants and the 11 Moroccan plants; all cross-validation, prediction, stability and calibration result tables; and the Supplementary Materials. The code is additionally mirrored in a public repository, https://github.com/htajmoua/medicinal_plants. (21 September 2026).

## Supplementary Materials

The following supporting information can be downloaded at: https://www.mdpi.com/article/10.3390/life16101616/s1, Table S1: Bootstrap distribution of the scores of the 15 unlabelled plants. The complete pipeline was refitted on 1000 bootstrap resamples of the 183 labelled records drawn with replacement, and every unlabelled plant was scored by each refitted model. The point estimate is the score of the model trained on all 183 records. The rank is computed among the 11 Moroccan plants only, from the highest score (rank 1) to the lowest (rank 11). Table S2: Predicted classes over the range of decision thresholds selected in the five cross-validation fold shuffles (0.30 to 0.44). For each threshold, the number of Moroccan plants and of validation plants called induction, the agreement of the validation calls with the reference labels, and the plants whose call differs from the call at the operating threshold t = 0.39. Table S3: Three nearest labelled records of each of the 15 unlabelled plants in the raw BiomedBERT embedding space (cosine similarity of the 768-dimensional mean-pooled vectors, before standardisation and PCA). Over all 15 × 183 pairs the cosine similarity ranges from 0.962 to 0.999, which reflects the strong anisotropy of the raw embedding space discussed in Section 3.2. Figure S1: (A) Bootstrap distribution of the scores of the 15 unlabelled plants (1000 resamples of the training set). Boxes show the interquartile range and the median, whiskers extend to the 2.5th and 97.5th percentiles, black diamonds are the point estimates of the model trained on all 183 records, the dashed line is the operating threshold t = 0.39 and the shaded band is the range of thresholds selected in the five cross-validation fold shuffles (0.30 to 0.44). Blue boxes, validation plants; orange boxes, Moroccan plants. (B) Reliability diagram of the out-of-fold probabilities obtained from ten repetitions of stratified 5-fold cross-validation on the 183 labelled records, in five quantile bins (n, number of records per bin). The dotted diagonal is perfect calibration. Brier score 0.179 (0.247 for a constant prediction at the class prior), calibration slope 1.60, expected calibration error 0.086.
