# Supplementary material for: Unveiling the Immunomodulatory Potential of Medicinal Plants: A Machine Learning Framework Coupled with Experimental Validation
Source: Life (Basel). 2026 Sep 26;16(10):1616. doi: 10.3390/life16101616 (PMC13705700; doi:10.3390/life16101616)
Supplement: Supplementary file 1 [file life-16-01616-s001.zip › life-4564777-supplementary.pdf]

Supplementary Materials

Unveiling the Immunomodulatory Potential of Medicinal Plants: A Machine Learning Framework Coupled with Experimental Validation

Aicha Assouab, Hamza Tajmouati, Fatima Abdou-Allah and Khadija Akarid

This document contains Tables S1 to S3 and Figure S1 referred to in the main text. All values were produced by the scripts deposited with the dataset (Zenodo, https://doi.org/10.5281/zenodo.19451645). The model is the logistic regression (C = 0.001, L2 penalty) on BiomedBERT embeddings after standardisation and PCA at 95% explained variance, trained on the 183 labelled records, with the decision threshold t = 0.39; P denotes the model score P(class = 1, induction).

Table S1. Bootstrap distribution of the scores of the 15 unlabelled plants. The complete pipeline was refitted on 1,000 bootstrap resamples of the 183 labelled records drawn with replacement, and every unlabelled plant was scored by each refitted model. The point estimate is the score of the model trained on all 183 records. The rank is computed among the 11 Moroccan plants only, from the highest score (rank 1) to the lowest (rank 11).

Set	Species	P (point estimate)	Bootstrap mean	Bootstrap SD	95% bootstrap interval	Induction calls at t = 0.39 (%)	Rank among the 11 Moroccan plants, mean (95% interval)
Validation	*Kalanchoe pinnata*	0.765	0.764	0.052	0.654 to 0.854	100	n/a
Validation	*Phyllanthus amarus*	0.687	0.682	0.064	0.549 to 0.795	100	n/a
Moroccan	*Myrtus communis*	0.588	0.594	0.068	0.454 to 0.721	100	1.2 (1 to 3)
Moroccan	*Salvia clandestina*	0.538	0.557	0.064	0.427 to 0.678	99.5	2.1 (1 to 4)
Moroccan	*Origanum elongatum*	0.458	0.473	0.082	0.314 to 0.640	83.9	4.4 (1 to 10)
Validation	*Eremanthus crotonoides*	0.437	0.444	0.069	0.318 to 0.582	76.7	n/a
Moroccan	*Capparis spinosa*	0.414	0.431	0.082	0.273 to 0.597	68.9	5.6 (2 to 10)
Moroccan	*Origanum compactum*	0.409	0.424	0.064	0.305 to 0.550	67.8	5.7 (3 to 10)
Moroccan	*Warionia saharae*	0.406	0.421	0.070	0.295 to 0.566	64.5	5.7 (3 to 9)
Moroccan	*Ephedra altissima*	0.394	0.404	0.067	0.285 to 0.537	57.9	6.6 (3 to 10)
Moroccan	*Euphorbia echinus*	0.387	0.401	0.075	0.257 to 0.564	55.3	6.7 (3 to 10)
Validation	*Empetrum nigrum L.*	0.376	0.388	0.064	0.271 to 0.524	48	n/a
Moroccan	*Ephedra fragilis*	0.352	0.374	0.073	0.238 to 0.540	37.8	8.2 (4 to 11)
Moroccan	*Lavandula dentata*	0.345	0.364	0.065	0.252 to 0.497	34.4	8.7 (6 to 10)
Moroccan	*Cannabis sativa*	0.215	0.228	0.055	0.139 to 0.359	0.9	10.9 (9 to 11)

SD, standard deviation over the 1,000 resamples; the 95% interval is delimited by the 2.5th and 97.5th percentiles. Induction calls is the percentage of resamples in which the score was at least 0.39. n/a, not applicable (validation plants are not ranked with the Moroccan set).

Table S2. Predicted classes over the range of decision thresholds selected in the five cross-validation fold shuffles (0.30 to 0.44). For each threshold, the number of Moroccan plants and of validation plants called induction, the agreement of the validation calls with the reference labels, and the plants whose call differs from the call at the operating threshold t = 0.39.

Threshold t	Moroccan plants called induction	Validation plants called induction	Validation agreement with reference labels	Plants whose call differs from the call at t = 0.39
0.30	10 of 11	4 of 4	2 of 4	*Empetrum nigrum L., Ephedra fragilis, Lavandula dentata, Euphorbia echinus*
0.31	10 of 11	4 of 4	2 of 4	*Empetrum nigrum L., Ephedra fragilis, Lavandula dentata, Euphorbia echinus*
0.32	10 of 11	4 of 4	2 of 4	*Empetrum nigrum L., Ephedra fragilis, Lavandula dentata, Euphorbia echinus*
0.33	10 of 11	4 of 4	2 of 4	*Empetrum nigrum L., Ephedra fragilis, Lavandula dentata, Euphorbia echinus*
0.34	10 of 11	4 of 4	2 of 4	*Empetrum nigrum L., Ephedra fragilis, Lavandula dentata, Euphorbia echinus*
0.35	9 of 11	4 of 4	2 of 4	*Empetrum nigrum L., Ephedra fragilis, Euphorbia echinus*
0.36	8 of 11	4 of 4	2 of 4	*Empetrum nigrum L., Euphorbia echinus*
0.37	8 of 11	4 of 4	2 of 4	*Empetrum nigrum L., Euphorbia echinus*
0.38	8 of 11	3 of 4	3 of 4	*Euphorbia echinus*
0.39	7 of 11	3 of 4	3 of 4	none
0.40	6 of 11	3 of 4	3 of 4	*Ephedra altissima*
0.41	4 of 11	3 of 4	3 of 4	*Warionia saharae, Ephedra altissima, Origanum compactum*
0.42	3 of 11	3 of 4	3 of 4	*Warionia saharae, Ephedra altissima, Capparis spinosa, Origanum compactum*
0.43	3 of 11	3 of 4	3 of 4	*Warionia saharae, Ephedra altissima, Capparis spinosa, Origanum compactum*
0.44	3 of 11	2 of 4	4 of 4	*Eremanthus crotonoides, Warionia saharae, Ephedra altissima, Capparis spinosa, Origanum compactum*

The five fold shuffles returned optimal thresholds of 0.38, 0.39, 0.44, 0.30 and 0.43 (mean 0.39). Only *M. communis*, *S. clandestina*, *O. elongatum*, *C. sativa*, *K. pinnata* and *P. amarus* keep the same call over the whole range.

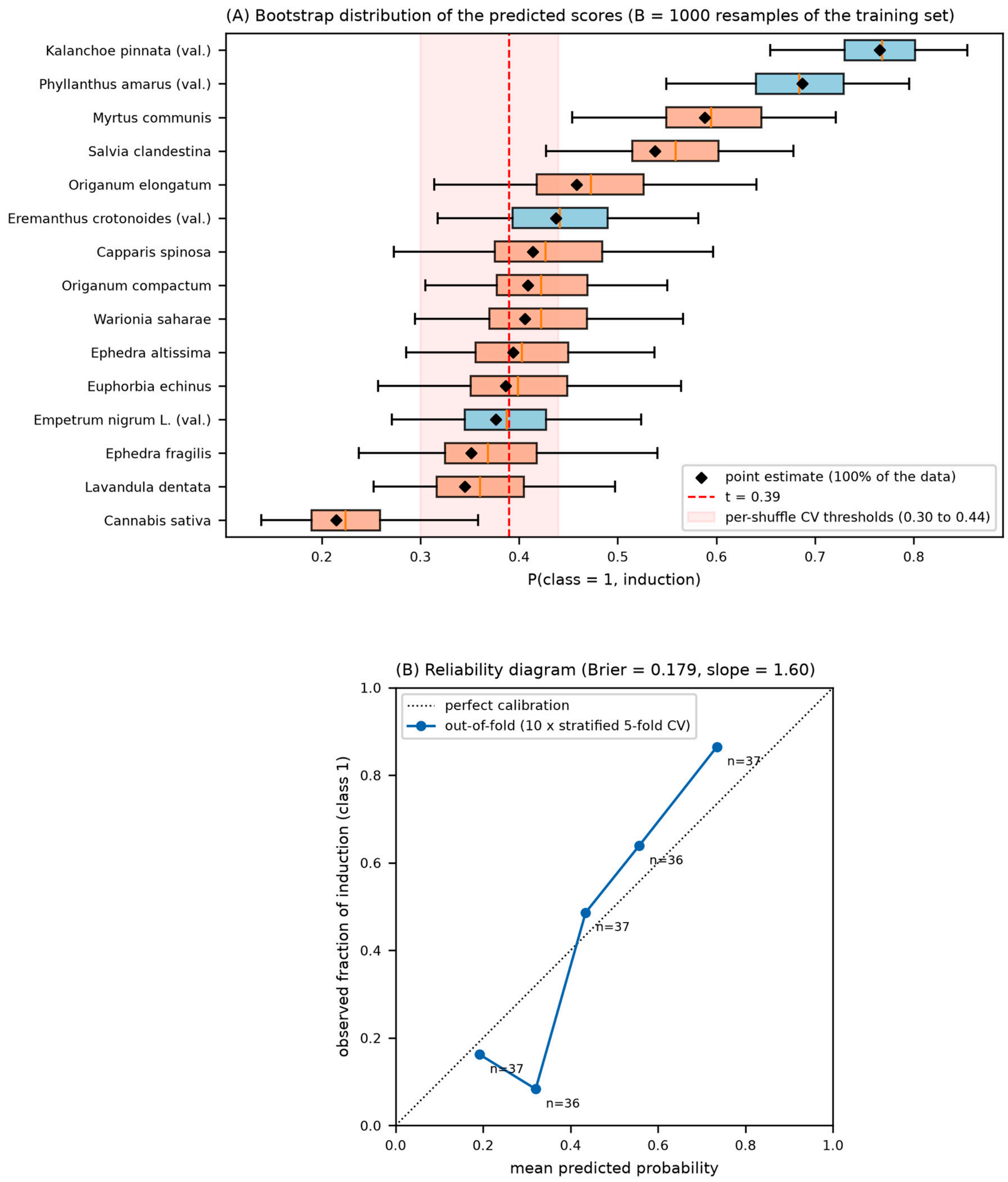

Figure S1. (A) Bootstrap distribution of the scores of the 15 unlabelled plants (1,000 resamples of the training set). Boxes show the interquartile range and the median, whiskers extend to the 2.5th and 97.5th percentiles, black diamonds are the point estimates of the model trained on all 183 records, the dashed line is the operating threshold t = 0.39 and the shaded band is the range of thresholds selected in the five cross-validation fold shuffles (0.30 to 0.44). Blue boxes, validation plants; orange boxes, Moroccan plants. (B) Reliability diagram of the out-of-fold probabilities obtained from ten repetitions of stratified 5-fold cross-validation on the 183 labelled records, in five quantile bins (n, number of records per bin). The dotted diagonal is perfect calibration. Brier score 0.179 (0.247 for a constant prediction at the class prior), calibration slope 1.60, expected calibration error 0.086.

Table S3. Three nearest labelled records of each of the 15 unlabelled plants in the raw BiomedBERT embedding space (cosine similarity of the 768-dimensional mean-pooled vectors, before standardisation and PCA). Over all 15 x 183 pairs the cosine similarity ranges from 0.962 to 0.999, which reflects the strong anisotropy of the raw embedding space discussed in Section 3.2.

Set	Unlabelled plant	P	Rank	Nearest labelled record	Label	Region	Used part	Metabolite content (truncated)	Tested microorganism (truncated)	Cosine similarity
Validation	*Kalanchoe pinnata*	0.765	1	*Chenopodium ambrosioides*	inhibition	BRAZIL	AERIAL PART	Kaempferol phenol	parasite	0.996
Validation	*Kalanchoe pinnata*	0.765	2	*Solanum lycocarpum*	induction	BRAZIL	fruit	alkaloid	Schistosoma mansoni parasite	0.996
Validation	*Kalanchoe pinnata*	0.765	3	*potato tuber*	induction	INDIA	TUBERCULE	ND	Leishmania donovani	0.996
Validation	*Phyllanthus amarus*	0.687	1	*Psidium guajava*	inhibition	INDIA	LEAF	quercetin,phenols, alkaloids, carbohydrates and steroids as	Escherichia coli (EPEC) [MTCC-724]	0.996
Validation	*Phyllanthus amarus*	0.687	2	*Piper nigrum*	induction	india	seed	piperine terpene	Leishmania donovani culture (MHOM/IN/83/AG83)	0.995
Validation	*Phyllanthus amarus*	0.687	3	*Chenopodium ambrosioides*	inhibition	BRAZIL	AERIAL PART	Kaempferol phenol	parasite	0.995
Validation	*Empetrum nigrum L.*	0.376	1	*Eucommia ulmoides*	inhibition	CHINA	FLOWER	ND	ND	0.997
Validation	*Empetrum nigrum L.*	0.376	2	*Smilax glabra*	induction	Korea	root	polysaccharides	ND	0.996
Validation	*Empetrum nigrum L.*	0.376	3	*Chasmanthera dependens*	induction	KENYA	STEM BARK_LEAF	berberine type alkaloids	L.Major	0.996
Validation	*Eremanthus crotonoides*	0.437	1	*Kielmeyera membranacea*	inhibition	BRAZIL	leaf(Dichloromethane)	triterpene	t Mycobacterium tuberculosis H37Rv (ATCC 2729	0.999
Validation	*Eremanthus crotonoides*	0.437	2	*Apium graveolens*	inhibition	ITALY	LEAF	apiin phenol	ND (microorganism) but tested on J774cells	0.995
Validation	*Eremanthus crotonoides*	0.437	3	*Echium amoenum*	induction	IRAN	Flowers	phenol, alkaloid and terpene (main component)	L. major strain MRHO/IR/75/ER	0.995
Moroccan	*Warionia saharae*	0.406	1	*Rhus tripartitum*	inhibition	TUNISIA	root barks	phenol	Staphylococcus aureus	0.995
Moroccan	*Warionia saharae*	0.406	2	*Scrophularia striata*	inhibition	IRAN	Aerial part	Flavonoid phenol	Staphylococcus aureus ATCC 25923, Staphylococ	0.995
Moroccan	*Warionia saharae*	0.406	3	*Gymnosporia senegalensis*	inhibition	SOUTH AFRICA	LEAF	alkaloid phenol terpen steroid	Mycobacterium tuberculosis bacteria	0.995
Moroccan	*Ephedra altissima*	0.394	1	*Rhus tripartitum*	inhibition	TUNISIA	root barks	phenol	Staphylococcus aureus	0.995
Moroccan	*Ephedra altissima*	0.394	2	*Scrophularia striata*	inhibition	IRAN	Aerial part	Flavonoid phenol	Staphylococcus aureus ATCC 25923, Staphylococ	0.995
Moroccan	*Ephedra altissima*	0.394	3	*Gymnosporia senegalensis*	inhibition	SOUTH AFRICA	LEAF	alkaloid phenol terpen steroid	Mycobacterium tuberculosis bacteria	0.994
Moroccan	*Ephedra fragilis*	0.352	1	*Gynura procumbens*	inhibition	MALAYSIA	WHOLE PLANT	ALKALOID_PHENOL_sterol	ND	0.995
Moroccan	*Ephedra fragilis*	0.352	2	*Limoniastrum guyonianum*	induction	TUNISIA	GALL	flavonoids, tannins and polyphenols phenol	ND	0.995
Moroccan	*Ephedra fragilis*	0.352	3	*Eucommia ulmoides*	inhibition	CHINA	FLOWER	ND	ND	0.995
Moroccan	*Capparis spinosa*	0.414	1	*Gynura procumbens*	inhibition	MALAYSIA	WHOLE PLANT	ALKALOID_PHENOL_sterol	ND	0.996
Moroccan	*Capparis spinosa*	0.414	2	*Moringa oleifera*	inhibition	Thailand	POD_SEED	ND	ND	0.995
Moroccan	*Capparis spinosa*	0.414	3	*Artemisia dracunculus*	induction	IRAN	ND	artemisinin: Terpen_ALKALOID_PHENOL	Leishmania major	0.995
Moroccan	*Salvia clandestina*	0.538	1	*Rhus tripartitum*	inhibition	TUNISIA	root barks	phenol	Staphylococcus aureus	0.996
Moroccan	*Salvia clandestina*	0.538	2	*Gymnosporia senegalensis*	inhibition	SOUTH AFRICA	LEAF	alkaloid phenol terpen steroid	Mycobacterium tuberculosis bacteria	0.995
Moroccan	*Salvia clandestina*	0.538	3	*Larrea divaricata*	induction	ARGENTINA	LEAF_BRANCHE	ND	Mycobacterium tuberculosis	0.995
Moroccan	*Origanum compactum*	0.409	1	*Rhus tripartitum*	inhibition	TUNISIA	root barks	phenol	Staphylococcus aureus	0.995

Set	Unlabelled plant	P	Rank	Nearest labelled record	Label	Region	Used part	Metabolite content (truncated)	Tested microorganism (truncated)	Cosine similarity
Moroccan	*Origanum compactum*	0.409	2	*Gymnosporia senegalensis*	inhibition	SOUTH AFRICA	LEAF	alkaloid phenol terpen steroid	Mycobacterium tuberculosis bacteria	0.995
Moroccan	*Origanum compactum*	0.409	3	*Echium amoenum*	induction	IRAN	Flowers	phenol, alkaloid and terpene (main component)	L. major strain MRHO/IR/75/ER	0.995
Moroccan	*Lavandula dentata*	0.345	1	*Scrophularia striata*	inhibition	IRAN	Aerial part	Flavonoid phenol	Staphylococcus aureus ATCC 25923, Staphylococ	0.995
Moroccan	*Lavandula dentata*	0.345	2	*Gymnosporia senegalensis*	inhibition	SOUTH AFRICA	LEAF	alkaloid phenol terpen steroid	Mycobacterium tuberculosis bacteria	0.995
Moroccan	*Lavandula dentata*	0.345	3	*Kielmeyera membranacea*	inhibition	BRAZIL	leaf(Dichloromethane)	triterpene	t Mycobacterium tuberculosis H37Rv (ATCC 2729	0.995
Moroccan	*Origanum elongatum*	0.458	1	*Protium paniculatum var. modestum (PPM*	inhibition	BRAZIL	oleoresins EO	Triterpenes	nd	0.992
Moroccan	*Origanum elongatum*	0.458	2	*Psiadia punctulata*	induction	KENYA	STEM BARK_LEAF	flavones and phenyl propenoids phenol	L.Major	0.992
Moroccan	*Origanum elongatum*	0.458	3	*Pedilanthus tithymaloides*	inhibition	india	leaf	luteolin phenol	Herpes Simplex Virus (HSV)HSV-2G (ATCC734) Vi	0.992
Moroccan	*Myrtus communis*	0.588	1	*Rhus tripartitum*	inhibition	TUNISIA	root barks	phenol	Staphylococcus aureus	0.996
Moroccan	*Myrtus communis*	0.588	2	*Morinda citrifolia*	induction	BRAZIL	FRUIT	phenolic compound	Leishmania amazonensis	0.996
Moroccan	*Myrtus communis*	0.588	3	*Larrea divaricata*	induction	ARGENTINA	LEAF_BRANCHE	ND	Mycobacterium tuberculosis	0.996
Moroccan	*Euphorbia echinus*	0.387	1	*Cinnamomum camphora*	inhibition	Korea	LEAF	camphor terpene alkaloid	ND	0.995
Moroccan	*Euphorbia echinus*	0.387	2	*Larrea divaricata*	induction	ARGENTINA	LEAF_BRANCHE	ND	Mycobacterium tuberculosis	0.994
Moroccan	*Euphorbia echinus*	0.387	3	*Eucommia ulmoides*	inhibition	CHINA	FLOWER	ND	ND	0.994
Moroccan	*Cannabis sativa*	0.215	1	*Santolina impressa*	inhibition	Portugal	stems	monoterpene hydrocarbons and oxygenated monoterpenes, β-pin	C.albicans fungus	0.995
Moroccan	*Cannabis sativa*	0.215	2	*Laurencia Dendroidea*	inhibition	BRAZIL	WHOLE PLANT	l (1), obtusol (2), and cartilagineol terpene	Mycobacterium tuberculosis.bacteria	0.995
Moroccan	*Cannabis sativa*	0.215	3	*Anthemis wiedemanniana*	inhibition	TURKEY	Aerial parts	sesquiterpenes (10.1%). The presence of irregular monoterpen	Bacillus cereus (ATCC 11778) 50 50 1.56 Bacil	0.995

Label, class of the labelled record (induction = class 1, inhibition = class 0). Metabolite content and tested microorganism are reproduced from the source workbook and truncated for display; the full strings are in the deposited dataset.
